# A new method for the analysis of access period experiments, illustrated with whitefly-borne cassava mosaic begomovirus

**DOI:** 10.1371/journal.pcbi.1011291

**Published:** 2023-08-10

**Authors:** Ruairí Donnelly, Christopher A. Gilligan

**Affiliations:** Department of Plant Sciences, University of Cambridge, Cambridge, United Kingdom; Fundação Getúlio Vargas: Fundacao Getulio Vargas, BRAZIL

## Abstract

Reports of low transmission efficiency, of a cassava mosaic begomovirus (CMB) in *Bemisia tabaci* whitefly, diminished the perceived importance of whitefly in CMB epidemics. Studies indicating synergies between *B. tabaci* and CMB prompt a reconsideration of this assessment. In this paper, we analysed the retention period and infectiousness of CMB-carrying *B. tabaci* as well as *B. tabaci* susceptibility to CMB. We assessed the role of low laboratory insect survival in historic reports of a 9d virus retention period. To do this, we introduced Bayesian analyses to an important class of experiment in plant pathology. We were unable to reject a null hypothesis of life-long CMB retention when we accounted for low insect survival. Our analysis confirmed low insect survival, with insects surviving on average for around three days of transfers from the original infected plant to subsequent test plants. Use of the new analysis to account for insect death may lead to re-calibration of retention periods for other important insect-borne plant pathogens. In addition, we showed that *B. tabaci* susceptibility to CMB is substantially higher than previously thought. We also introduced a technique for high resolution analysis of retention period, showing that B. tabaci infectiousness with CMB was increasing over the first five days of infection.

## Author summary

One of the major crops for food security is cassava. Diseases caused by whitefly-borne viruses, such as cassava mosaic begomoviruses (CMBs; causative agent of cassava mosaic disease), are among the major constraints of cassava production in sub-Saharan Africa. Reports of relatively low transmission efficiency of CMB in *Bemisia tabaci* whitefly helped to diminish the perceived importance of whitefly in CMB epidemics. But what role has the rate of insect laboratory survival played in these reports? Here we introduced Bayesian methods of statistical analysis to account for insect laboratory survival in the analysis of an important class of experiment in plant pathology. In essence, we show, for CMB, that published laboratory data is consistent with high insect transmission efficiency and low insect laboratory survival. We also show for CMB that there is no evidence that the retention period—the length of time the pathogen-bearing insect vector is infectious—is less than the insect’s lifespan. Our methods can be used to analyse published laboratory data for a range of additional insect-borne plant pathogens, and, in particular, to analyse viral retention period—a central parameter in the classification of viruses according to their transmission type.

## Introduction

Laboratory-based plant pathologists often measure the frequency of plant infection when plants are challenged by cohorts of insect vectors that have previously been exposed to plant pathogens. This type of experiment is sometimes referred to as an access period experiment [1] (AP), because of an emphasis on the duration of the insect’s access to a host plant—see example studies [2–5]. One variant of the AP experiment has sought to analyse the pathogen’s retention period, i.e., the length of time the pathogen-bearing insect vector is infectious, using a sequence of access periods, but this experimental variant may have been prone to a confounding variable, low laboratory insect survival. In this paper we bring modern statistical methods—that allow artefacts to be explicitly modelled—to bear on the analysis of AP experiments. In the widely-cited study of Dubern [2], the total length of a sequence of inoculation access periods was varied leading to a conclusion that cassava mosaic begomovirus (CMB) is retained by the *Bemisia tabaci* whitefly vector for 9 days. The original Dubern [2] study reported that this estimate may have been influenced by low insect survival. It is possible that the retention period estimate for a range of insect-borne plant and animal pathogens were also influenced by low insect survival. In this paper, we clarify what the Dubern [2] data mean for CMB retention when low insect survival is taken into account. We also assess *B. tabaci* susceptibility (the proportion of insects that successfully acquire the pathogen) and *B. tabaci* infectiousness with CMB (the proportion of plants that are successfully inoculated by a pathogen-carrying insect).

Cassava mosaic begomovirus (henceforth, CMB) refers to various species of a cassava begomovirus species complex from the geminivirus family. CMB has been present in sub-Saharan Africa for more than a century [6]—but re-emerged as a particularly severe infection of cassava in east-Africa in the 1990’s [7], which is still spreading regionally [8, 9]. Transmission by infected plant cuttings is an established means of CMB spread in cassava—in addition to *B. tabaci*-borne transmission. In many locales, inspection of CMB-infected cassava plants has indicated that the majority of plant infections have arisen through contaminated cuttings [10, 11]. This has led to a perception in the field that transmission via insects is not as important as transmission via infected cuttings (i.e., a cuttings-centric view of CMB epidemics), see [10] and Kenya subsection of Legg and Thresh [12]. This cuttings-centric view was supported by findings that appeared to demonstrate low CMB transmission efficiency in *B. tabaci* [2] (see eg. CABI [13]). It should be noted, however, that in the absence of preferential selection of infected material for plant cuttings, initial disease incursions are likely to fade out if the only means of viral spread is in infected plant cuttings. This is because propagation without preferential selection of infected material can at most lead to the persistence of a background level of incidence in the new season i.e. no epidemic growth. Furthermore, elevated *B. tabaci* abundance has been repeatedly observed in combination with severely diseased cassava [14]—and this has been linked to a synergistic interaction between the vector and the pathogen [15–19]. These considerations mean that a reappraisal of the transmission efficiency of CMB-carrying whitefly is now timely. The present work contributes to the reappraisal by applying modern Bayesian methods of data analysis to the original laboratory dataset that appeared to conclude low transmission efficiency of *African cassava mosaic virus* in *B. tabaci*.

In AP experiments, cohorts of insects are exposed to pathogen-infected plants (acquisition access). The resulting potentially pathogen-bearing insects are exposed to healthy test plants (inoculation access). In the common retention period variant of this experiment, insect cohorts undergo a sequence of transfers with separate inoculation periods on a sequence of healthy plants. Consider the last plant in the sequence of test plants that becomes infected in any of the replicates—here referred to as the ‘terminal index of infection’. The retention period has been estimated with the terminal index of infection in a number of studies including Dubern [2] (see also e.g., Simmons et al. [3] and Barajas-Ortiz et al. [4]). There may be serious flaws, however, in the identification of the terminal index of infection with retention estimates (e.g. the corresponding 9d retention estimate in Dubern [2]). Firstly, the terminal index of infection is likely to increase if more replicates are performed. Had, for instance, Dubern [2] used more replicates, then the terminal index of CMB infection would be ≥ 9. Since the expected response depends on the number of replicates, but the underlying retention period does not, this implies bias in the estimate (the expected value of an unbiased estimator and the true value of the parameter being estimated should be equal with only the uncertainty associated with the expected response changing with the number of replicates). Secondly, the number of live insects at each successive inoculation access period will tend to decline due to mortality. In summary, the nature of the existing CMB retention period estimate, and the lack of accounting for insect survival, are liable to have involved experimental bias (e.g., omitted-variable bias [20]).

When assessing the results of experiments it is important to take laboratory artefacts like low insect survival into account and to avoid systematic sources of bias. Model-based Bayesian analyses offer one way to do this. By model-based Bayesian analysis we mean the derivation of probability models for fitting to experimental data in order to make statistical inferences. Inferences include parameter estimation for parameters relating to the response of interest and to additional confounding processes. In this paper, we introduce model-based Bayesian analysis to a classic experiment that is common in plant pathology. Our specific goal is to clarify what a widely-cited dataset [2] actually says concerning CMB retention in *B. tabaci*. Retention estimates are important for several reasons. They are used by plant pathologists to characterise the transmission type of a pathogen, and by epidemiologists to predict epidemic trajectories. We also introduce a new, high resolution, technique for analysing retention period. For each day of the retention period, the method produces snapshots of insect infectiousness—illustrated here for the case of CMB retention by *B. tabaci* whitefly.

## Methods

### Experiment setup

**Fig 1:**
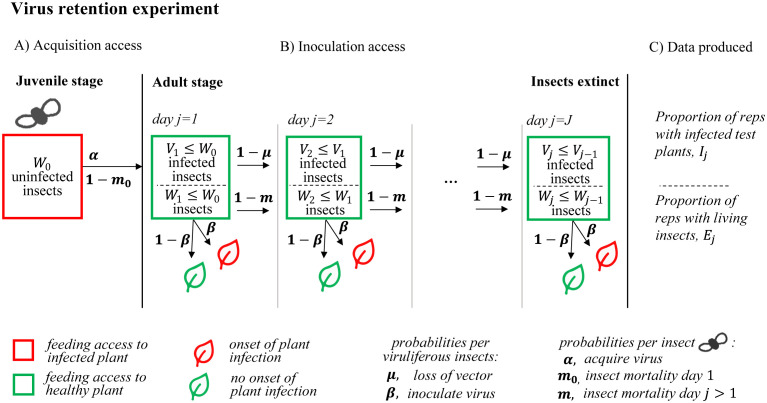
Schematic description of the retention period assay reported in Dubern [2]. Retention period assays include an acquisition (A) and at least one inoculation (B) access period (AAP and IAP respectively), and produce summary data (C). In Dubern [2] the IAP sequence was composed of a total of 13, i.e., *J* = 13, day-long exposures of *B. tabaci* cohorts to a sequence of healthy cassava test plants. The two components of data that were recorded were the number of replicate cohorts per *j*^*th*^ IAP, for *j* = 1..*J*: having live insects (*E*_*j*_, ‘number of inoculated plants’, Table 1 row 1), and leading to infected plants (*I*_*j*_, ‘number of infected plants’, Table 1 row 2). The patterns in the data that were recorded depend on two unobserved random variables: the number of alive insets per cohort per IAP (denoted *W*_*j*_, legend) and the number of alive infected insects per cohort per IAP (denoted *V*_*j*_ ≤ *W*_*j*_, legend). The probability model that corresponds to the schematic involves changes in the unobserved random variables due to virus acquisition and inoculation (*α* and *β* respectively, see legend), and for serial infected insect loss (*μ*, see legend) as well as for initial and serial insect mortality (*m*_0_, *m* respectively, see legend). See S1 Appendix for model details.

The retention period assay of Dubern [2] is an example of a generic set of experiments in plant and animal pathology of insect-borne pathogens (Fig 1). In the retention period assay of Dubern [2], groups of *B. tabaci* whitefly nymphs were raised on cassava plants infected with CMB (acquisition access period, Fig 1A, henceforth, AAP). Cohorts (*W*_0_) of potentially infected whitefly were then transferred for daily inoculation access periods (henceforth, IAP) to successive healthy plants up to cohort extinction (inoculation access, Fig 1B). Dubern [2] used *W*_0_ = 10 with 30 replicates and performed daily cohort transfers until all replicate insect cohorts became extinct (this corresponded to a maximum of 12 daily transfers for a replicate cohort). The dataset comprised, for each daily transfer, the number of replicates in which the insect population was extant (reproduced in Table 1 as ‘live cohort’, denoted *E*_*j*_) and the number of replicates in which infection of the healthy test plant ensued (reproduced in Table 1 as ‘plant infection’, denoted *I*_*j*_); see also Fig 1C for ‘Data produced’.

**Table 1:** Empirical data from the retention period assay described in Dubern [2]. The investigator recorded two components of data: the ‘number of inoculated plants’ (which we describe as the number of replicate cohorts having live insects)(*E*_*j*_ row 1), and the ‘number of infected plants’ (*I*_*j*_, row 2). Both components were recorded for the *j*^*th*^ day-long IAP for each of a total of 13 consecutive IAPs (header row) and with each component corresponding to a total of 30 replicates. The setup involved consecutive daily transfers of insect cohorts for each experimental replicate. Throughout the text, we accordingly refer to either the *j*^*th*^ daily transfer or IAP, as appropriate. Note that if the presence of infected insects was not limited by mortality, then the final transfer day for which plant inoculation occurred (row 2, bold) would reflect retention period of the pathogen to some degree. Since insect survival was an important factor, however, it is possible that the same measure reflects mainly the tendency for infected insects to be lost from the experiment due to manual transfer associated mortality.

*Successive transfers*	*0*	*1*	*2*	*3*	*4*	*5*	*6*	*7*	*8*	*9*	*10*	*11*	*12*
**E**_**j**_	30	30	27	23	22	19	15	12	8	7	5	3	1
**I**_**j**_	28	24	20	20	19	12	8	3	3	**1**	0	0	0

### Data analysis

We analysed the Dubern [2] data by deriving two simple probability models of the experiment. The models are equivalent but present different perspectives on the same problem. We present a very brief overview of each model below. The full details are given in S1–S4 Appendices, including non-technical as well as technical descriptions. Both models account for the four processes that drive transmission in the laboratory and their impact on the insect population variables (nb. these variables are were not observed in the experiment and appear in the model as latent variables). The processes (and associated model parameters) are insect mortality (*m*), loss of insect infectiousness (*μ*), as well as virus acquisition (*α*) and inoculation/insect infectiousness (*β*).

Fig 1 illustrates how random variables change in proportion to probability parameters as the number of cohort transfers increases. The strategy is to find the unknown parameter values that best fit the observed data (reproduced in Table 1). We use Bayesian model fitting with an MCMC algorithm to combine model 1 or model 2 and the summary data in Table 1. Bayesian inference involves the use of data (or evidence) to update a prior. In this case the data comprise the observations recorded in the Dubern [2] experiment and the prior distribution for the parameters is chosen to be non-informative but of an appropriate distribution family given the nature of the underlying parameters (i.e., *beta*(1, 1) prior distributions, which are equal to the non-informative uniform distribution on the [0, 1] interval).

#### Retention

Model 1 is comprised of equations for the probability that an insect cohort is alive and for the probability that a test plant is infected. Both equations are formulated for the general case of the *j*^*th*^ IAP. The analysis of model 1 provides an inference for the death rate of insects *m*. It also provides an inference for the rate of loss of infectiousness from infected insects (*μ*) which may occur through death or through pathogen clearance (additional parameters inferred in model 1 are described in S1 Appendix). The parameter fits for *m* and *μ*, in the form of parameter distributions, are then combined using MCMC to form a test statistic. The test statistic is used to test the null hypothesis that there is no evidence for *B. tabaci* clearance of CMB. In other words, we test the null hypothesis that CMB is retained for life by *B. tabaci*. The details of Model 1 and of the hypothesis test are given in S1 and S2 Appendices respectively.

#### Susceptibility and infectiousness

Note that we make a distinction in terminology between *insect susceptibility* and *insect infectiousness*. By insect susceptibility we mean the propensity for insect vectors to acquire the pathogen when feeding on infected plants (this corresponds to the probability of pathogen acquisition in the AAP, denoted *α*). By insect infectiousness we mean the propensity for infected insect vectors to inoculate the pathogen when feeding on healthy plants (this corresponds to the probability of pathogen inoculation in the IAP, denoted *β*). In AP experiments the role of the initial pathogen acquisition step in overall transmission between plants (corresponding to insect susceptibility) cannot be separated from the subsequent plant inoculation step (corresponding to insect infectiousness), and vice-versa. This leads to the dilemma that AP experimenters cannot normally evaluate susceptibility and infectiousness—though this may be their chief concern (we refer to this as the AP dilemma). Instead, experimenters investigate these processes obliquely—by showing, for example, how overall transmission changes as the length of the AAP or the IAP is varied. It is not clear, however, that such patterns reflect only susceptibility (as the length of the AAP is varied) or infectiousness (as the length of the IAP is varied). A secondary aim of this paper is to additionally comment on insect infectiousness and insect susceptibility, to complement our investigation of retention period. We first illustrate the AP dilemma in Box 1, and we relate the AP dilemma to the statistical concept of identifiability using a basic model of the AP experiment in Box 1A. To explain the dilemma, for simplicity in Box 1 we ignore insect survival which is considered for retention period assays in the main text. Though the lack of identifiability for acquisition and inoculation remains intractable, the perspective of identifiability leads to a novel approach to studying susceptibility and infectiousness (Box 1 Bi-ii). The approach requires a second model that is closely related to model 1 (see S3 Appendix for full description).

**Box 1. The access period dilemma**

In access period (AP) experiments vectors acquire a pathogen from an infected plant and inoculate healthy plants. Experimenters wish to quantify acquisition and inoculation—but record only infection of test plants (termed the *AP dilemma* here). We relate the AP dilemma to the statistical concept of identifiability below. This leads to a new approach to the dilemma.

A) Simple statistical description and identifiability

An AP assay begins with a fixed access period for a cohort of *W*_0_ insects on a single infected plant (i.e., acquisition access period, *AAP*). The cohort is then transferred to a single healthy test plant (i.e., inoculation access period, *IAP*). The probability of test plant infection, denoted *q*, is then,

$$\begin{array}{c} q=1-{\left(1-\alpha \beta \right)}^{W_{0}} \end{array}$$ (1)

In Eq 1, *α* and *β* are the probabilities of pathogen acquisition, and test plant inoculation (or equivalently insect infectiousness). The number of infected plants in *n* replicates of the experiment, denoted *X*, is then binomially distributed (denoted by ∼*B*) in proportion to *q*,

$$\begin{array}{c} X∼B(n,q) \end{array}$$ (2)

In the AP dilemma, inference of a distribution for the compound parameter (*αβ*) is straightforward by relating Eq 2 to a relevant dataset—but there is no way to infer insect infectivity (*α*), or infectiousness (*β*) from the compound parameter distribution. This is the problem of non-identifiability in statistics.

B) Retention period assays

For retention period assays, we modify Eq 1 to include a sequence of IAPs on healthy plants (i.e., *β*_*j*_ for *j* = 1..*J*). The problem of non-identifiability prevents separate inference of distributions for insect infectivity (*α*) and of insect infectiousness (for each IAP in the sequence of IAPs, *β*_*j*_). Nevertheless, if we use a proxy inference, it may yet be possible to infer related quantities. For instance, we must know *α* if we are to infer insect infectiousness over the IAP sequence. But the use of the peak vector efficiency proxy inference instead, denoted by *αβ*_*peak*_, leads to related inferences for *α* and *β* (denoted by $\hat{\beta }$),

*(i) Insect infectiousness.* Using the inference of the proxy distribution, it is possible to infer distributions for the percentage peak infectiousness for each daily IAP *j*, i.e., $\hat{\beta_{j}}=\beta_{j}/\beta_{peak}$
*(ii) Insect infectivity.* An additional use of the peak vector efficiency inference is the deduction of a lower bound for infectivity, i.e., since *β*_*peak*_ ≤ 1 it follows that *α* ≥ *αβ*_*peak*_

See main text for further details on the use of the above related inferences.

Model 2 comprises a single equation for the probability of plant infection on the *j*^*th*^ IAP. The simplification relative to model 1 (i.e., 1 equation per daily IAP in model 2 compared with 2 equations per daily IAP in model 1) is achieved by excluding the process of pathogen clearance. The focus of model 2, instead, is a separate rate of plant inoculation (which represents insect infectiousness) for each daily IAP (*β*_*j*_). In this way, pathogen clearance is present implicitly in model 2 through the possibility of zero-infectiousness on a given daily IAP (cf. model 1). One advantage of model 2 is that it becomes possible to isolate information closely related to the inoculation probability from the acquisition probability—though not the inoculation probability per se. In the case of infectiousness, the process results in a set of inferences for percentage peak insect infectiousness for each daily IAP (Box 1 Bi). In the case of susceptibility, the process results in a lower bound for insect susceptibility (Box 1 Bii). Though a high resolution analysis (eg. model 2) may in general be preferable, in this work such an approach complements, rather than replaces, a lower resolution analysis (eg. model 1). At the most basic level we asked does the Dubern [2] dataset indicate rejection of a life-long viral retention null hypothesis for CMB. The corresponding hypothesis test requires parameter inference for the rate of loss of infectiousness from infected insects—which is only possible using model 1.

## Results

We describe the results of applying our methods to the published CMB dataset [2] in two parts. Results for the first part involve fitting probability model 1 to the data to estimate a pair of parameters: serial insect mortality, *m*, and, serial loss of infectiousness, *μ*. These parameters combine to form a test statistic with which to perform a hypothesis test (see subsection, *Insect retention*). The hypothesis test is the primary aim of this paper. Results for the second part involve using the simple methods outlined in Box 1 to isolate insect infectiousness according to the daily IAP. This provides a more detailed analysis of how insect infectiousness (to plants) varies over the course of an insect’s infection. We also estimate a lower bound for insect susceptibility.

### Insect retention

The estimated 95% credible intervals for the model 1 probabilities of initial transfer death (i.e, death in the first daily transfer for the first IAP) and serial transfer death (i.e, death in the *j*^*th*^ daily transfer or during the (*j* − 1)^*th*^ IAP) respectively were *m*_0_|_95%_ = [0.306, 0.768] and *m*|_95%_ = [0.228, 0.369] (Table 2a i-ii, with median values of ${\bar{m}}_{0}=0.487$ and $\bar{m}=0.297$ respectively)(both inferences were made using Eq S1.1 in S1 Appendix and the live cohort data, Table 1b). The estimated 95% credible intervals for the probability of serial loss of infectious insects was *μ*|_95%_ = [0.285, 0.374] (Table 2b i, with median value $\bar{\mu }=0.328$). The latter inferences were made using Eq S1.2 in S1 Appendix and the plant infection data, Table 2ii. An additional compound parameter that is not meaningful in its un-separated form but which is still needed for the modelling is referred to, for convenience, as vector efficiency, *αβ* (Table 2b iii). See Table A in S1 Appendix for a summary of model 1 parameters.

**Table 2:** Parameter estimates and hypothesis testing for data from a retention period assay using a model of insect vector infectiousness (Model 1, S1 Appendix, see also Fig 1 for assay schematic). Note that the model of insect vector infectiousness involved clearance of the pathogen by the insect and constant infectiousness up to clearance. In *a*), mortality probabilities were estimated from combining summary data representing cohorts that remain extant (see Table 1 row 1 for data) with Model 1. In *b*) probabilities of loss of infected insect and vector efficiency were estimated from combining summary data representing test plant infection (Table 1 row 2) with Model 1. Further statistical analysis tested the null hypothesis that there was no loss of infectious insect vectors beyond that predicted by insect mortality in the laboratory. The hypothesis involved the test statistic in *c*), which was derived from the posterior distributions in *a*) and *b*) (see Methods section in main text for further description). Posterior distributions were calculated using rStan v2.21.0 [33], and Model 1 (S1 Appendix) and posterior distributions are summarised throughout *a* − *c* by 50^*th*^ (median), 2.5^*th*^ and 97.5^*th*^ percentiles. Note that here, and in all subsequent results tables, for simplicity figures are truncated to the third decimal point. All analyses were carried out in R version 3.63 [34]. Note that low insect survival in the experiment (c iii) was associated with a test statistic that did not significantly deviate from 1 (c i).

Model 1		*parameter*			*median*	*2.5%*	*97.5%*
*a*	*live cohort data*	*i)*	day 1 mortality,	*m_0_*	0.487	0.306	0.768
		*ii)*	day *j* > 1 mortality,	*m*	0.297	0.228	0.369
*b*	*plant infection data*	*i)*	infected vector loss,	*μ*	0.328	0.285	0.374
		*ii)*	vector efficiency,	*αβ*	0.578	0.345	0.910
*c*	*derived estimates*	*i)*	test statistic,	$\frac{\left(1-\mu \right)}{\left(1-m\right)}$	0.955	0.850	1.085
		*ii)*	serial insect survival,	$\frac{1}{m}$	3.360	2.702	4.378
		*iii)*	overall insect survival,	$m_{0}+\left(1-m_{0}\right)\frac{1}{m}$	2.070	1.472	2.895

**Fig 2:**
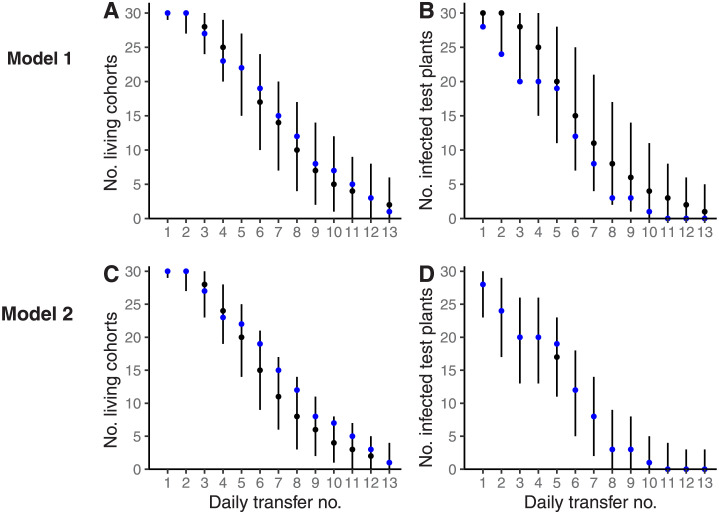
Empirical data-points from a retention period assay are reproduced by two models of insect infectiousness. The retention period assay described in Dubern [2] involved the cassava mosaic viral pathogen, cassava plants and the *B. tabaci* insect vector. In model 1 (A-B), infected insects are capable of clearing the pathogen, but are otherwise equally infectious to healthy plants regardless of IAP. In model 2 (C-D), infected insects are not capable of clearing the pathogen, but vary in infectiousness according to IAP (so that zero infectiousness corresponds to pathogen clearance). Median and 95% credible intervals for model assay output are represented throughout with black circles and vertical black bars respectively. Empirical observations from the Dubern [2] retention period assay are represented by blue circles. Posterior distributions were calculated using rStan v2.21.0 [33] and Model 1 or Model 2 (S1 Appendix and S3 Appendix) in combination with parameter estimates (see Tables 2 and 3 for summary); posterior distributions are summarised throughout *A* − *D* by 50^*th*^ (median, black circles), 2.5^*th*^ and 97.5^*th*^ percentiles (vertical black bars). All analyses were carried out in R version 3.63 [34].

We next assessed the model fits. When we combined the parameter inferences (Table 2a-b) with model 1 to generate synthetic data-points we found that they fit the assay data relatively well (Fig 2A and 2B). We note, however, a potential trend in the error for test plant infection in advance of day 5 (see subsequent section). Two further parameter distributions were then generated from the model 1 parameter fits. We estimated that the median lifespan of an insect in this part of the experiment was around 2.5 IAP days (this number accounts for a mortality associated with the first transfer day and a separate mortality associated with subsequent transfer days as set out in Table 2). For simplicity, we refer to this result as low *B. tabaci* laboratory survival. Secondly, we formed a test statistic from the ratio of the probability of insect death and infected insect loss, finding the 95% credible interval to be ((1 − *m*)/(1 − *μ*))|_95%_ = [0.85, 1.085]. When we considered, as a null hypothesis, the lifelong retention of CMB by *B. tabaci*, we were unable to reject the null hypothesis at the 95% confidence level—i.e., the 95% credible interval for (1 − *μ*)/(1 − *m*) comfortably includes 1 with a relatively small credible interval (± < *c*10% mean which we interpret as an indication of adequate statistical power).

### Insect infectiousness

The synthetic plant infection data generated from the model 1 parameter fits (Fig 2B) suggests minor overestimation of test plant infection prior to the 5^*th*^ IAP. One possibility is that the assumption of constant infectiousness of pathogen-bearing insects is not suitable. Accordingly, in model 2, insect infectiousness was allowed to vary by daily IAP (Model 2 and Box 1 Bi, percentage peak infectiousness approach). When we used Bayesian MCMC to fit probability model 2 to the data, the 95% credible intervals for the initial transfer death and for serial transfer death were *m*_0_|_95%_ = [0.327, 0.764] and *m*|_95%_ = [0.268, 0.388] respectively (Table 3 i-ii, with median values of ${\bar{m}}_{0}=0.497$ and $\bar{m}=0.329$ respectively). The estimated 95% credible interval for peak vector efficiency was *αβ*^*peak*^ = [0.721, 0.997] (Table 3 iii-iv, with median values of ${\bar{\alpha \beta }}^{peak}=0.931$). See Table B in S3 Appendix for a list of 95% credible intervals for the sequence of vector efficiencies that are specific to each daily IAP. All inferences were made using Eq S3.1 in S3 Appendix and the live cohort and plant infection data (Table 1a-b).

**Table 3:** Parameter estimates for data from a retention period assay using a modified model of insect vector infectiousness (Model 2, S3 Appendix). The model allowed insect vector infectiousness to vary across IAPs with zero infectiousness representing pathogen clearance. Mortality probabilities and peak vector efficiency were jointly estimated from summary data that comprised the number of cohorts that remained extant and test plants that became infected (Table 1) using Model 2. For additional posterior distributions representing percentage peak infectiousness and vector efficiency for each IAP, see Table B in S3 Appendix. See caption of Table 2 for further technical details.

Model 2	*parameter*			*median*	*2.5%*	*97.5%*
	*i)*	day 1 mortality,	*m_0_*	0.497	0.327	0.764
	*ii)*	day *j* > 1 mortality,	*m*	0.329	0.268	0.388
	*iii)*	vector efficiency,	*αβ^peak^*	0.931	0.721	0.997

**Fig 3:**
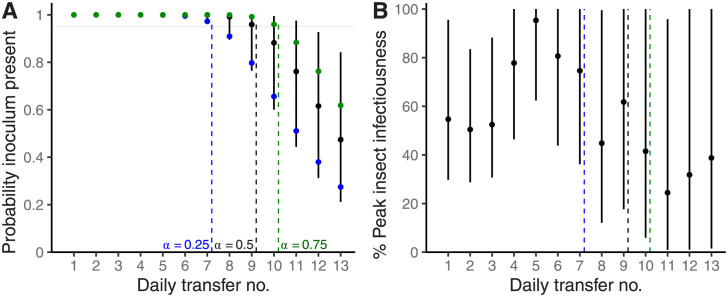
Insect infectiousness initially increases and experimental power rapidly diminishes, as the number of daily transfers increases in a retention period assay. For each daily IAP, we show: in A, the probability that any inoculum was present in the overall experiment (i.e., at least 1 infected insect in any replicate), and, in B, insect vector infectiousness to healthy plants (relative to peak insect infectiousness). In A, separate curves are included for representative probabilities of acquisition—see blue, black and green curves for *α* = 0.25, *α* = 0.5 and *α* = 0.75, respectively. In A and B, median and 95% credible intervals are represented with black circles and vertical black bars; for simplicity this is shown only for the representative case of *α* = 0.5 in A. Underlying results were generated in A and B using the parameter estimates in Table 2 (Model 1) and in Table 3 (Model 2), respectively. In A and B, a vertical black dashed line marks the daily IAP at which median probability of inoculum presence fell below the %95 confidence level marked by a red horizontal line in A (see also analogous blue and green vertical dashed lines for *α* = 0.25 and *α* = 0.75). See caption of Fig 2 for further technical description.

Scaling the sequence of vector efficiencies by peak vector efficiency leads to estimates for percentage peak infectiousness (Fig 3A; Box 1 Bi). From this set of deductions, it is apparent that peak infectiousness is not reached until around 5 days into adulthood/IAP. When we combined the parameter inferences (Table 3 i-ii and Table B in S3 Appendix) with Model 2 to generate synthetic data-points we found that they closely matched the frequency of test plant infection throughout the range (95% confidence intervals include the empirical data points; Fig 2C and 2D). See Table A in S3 Appendix for a summary of model 2 parameters.

### Insect susceptibility

Dubern [2] reported a range of 0.07 − 0.15 for the proportion of insects that acquire CMB, i.e., *B. tabaci* susceptibility. This finding was based on an additional component of the AP experiment, which we refer to as the transmission density assay. In the transmission density assay, adult insects in various cohort sizes that had been raised on CMB-infected plants were transferred to healthy cassava test plants where they were allowed to stay until death. It appears that Dubern [2] applied the formula $y=1-{\left(1-\delta \right)}^{W_{0}}$ to relate the proportion of plant infection to cohort sizes. Upon doing so, and following rearrangement of the equation, one can obtain estimates for *δ*. This operation produces the range 0.07 − 0.15 (Table 1 row 2 in Dubern [2]) for *B. tabaci* susceptibility (or, equivalently, *α* in our notation). There are a number of problems with this approach. As outlined in Box 1 and S1 Appendix, *δ* = *αβ*(1 − *m*_0_) (though note that in Box 1 we ignore insect survival for ease of presentation), therefore the method used in Dubern [2] implicitly assumes that *β* = 1 (100% virus inoculation per viruliferous insect) and *m*_0_ = 0 (zero insect mortality). Therefore the figures quoted for susceptibility in Dubern [2] can only be considered a lower bound (i.e., 0.07 < *α* < 1, or, equivalently, susceptibility may be anywhere from 7% to 100%). In addition, the range that is quoted did not take account of insect survival from manual transfer, nor that survival is likely to depend on the size of the cohorts (cf. cohorts ranged from 1–100 insects per plant so that reduced feeding/increased mortality is likely to occur at high densities compared with low densities).

We were unable to infer a distribution for susceptibility due to the lack of identifiability (see Box 1)—but we were able to provide a stricter lower bound on susceptibility using the peak infectiousness inference (*αβ*^*peak*^, Box 1). Given that *β*^*peak*^ ≤ 1, and a 95% credible interval for *αβ*^*peak*^ is [0.721, 0.997], we conclude that *α* > 0.721 (or, equivalently, susceptibility is likely to be greater than 70%). The lower susceptibility bound presented here indicates a substantially higher susceptibility than was indicated by Dubern [2] (in that the lower bound is around an order of magnitude bigger). Nevertheless it is important to realise that the lower bound presented here (≥ 70%) is consistent with the Dubern [2] susceptibility result if it is also interpreted as a lower bound.

## Discussion

The cassava mosaic geminivirus complex is known for its devastating impacts on human populations [21]. The combination of transmission through *B. tabaci* and plant cuttings has led to a high prevalence of severely infected cassava plants in sub-Saharan Africa [22, 23]. A perception has arisen in the field, in at least some regions, that the insect-borne mode of transmission is relatively unimportant compared with transmission through plant cuttings [11]. It is important to evaluate this perception. We have done this using modern statistical methods to analyse the parameters that have contributed to a view of low vector-borne transmission efficiency. Accordingly, the parameters that we evaluated in this paper were retention, infectiousness and susceptibility (of *B. tabaci* insect vectors with respect to CMB). Dubern [2] reported retention and susceptibility estimates for CMB in *B. tabaci*, but acknowledged the possible influence of low insect survival. When we analysed the Dubern [2] data accounting for low survival, a number of findings emerged. At the most basic level, the data did not support rejection of a null hypothesis of life-long CMB retention. Our results also show that insect infectiousness was rising for at least the initial portion of the retention period. In addition, the propensity for the virus to be acquired by the insect—i.e., insect susceptibility—was found to be substantially higher than was previously thought.

As a begomovirus, all of which are vectored by *B. tabaci*, CMB is thought to circulate, but not to replicate within the insect vector [24, 25]—i.e., it is a circulative non-popagative plant virus [25]. An exception to this is the *tomato yellow leaf curl* (TYLCV) begomovirus [26] that has been shown to replicate in *B. tabaci*. Since this indicates a circulative-propagative classification for TYLCV, questions remain as to whether additional begomoviruses may like TYLCV be considered candidates for whitefly pathogens [26, 27]. Propagative viruses are thought to be retained for the life of the insect vector, but this may not be the case for some non-propagative circulative viruses [25].

We framed our study in the following way. Firstly, given that the 9d retention estimate for CMB was likely to have been influenced by a confounding factor, could we show that retention is not life-long using the original dataset? This question led to a hypothesis test. Secondly, we asked were patterns apparent in how insect infectiousness waxes or wanes over the course of insect infection?

### No significant difference between B. tabaci survival- and infectious- periods

When we accounted for the confounding factor of low insect survival in the Dubern [2] data, we were not able to reject a null hypothesis of lifelong CMB retention. Indeed, low insect survival was confirmed with individual insects estimated to have survived only around three serial transfers on average (and around two transfer days overall when the mortality associated with the initial infected plant transfer was included, Table 2c iii). We note in particular that the number of experimental replicates with alive insect cohorts was predicted very successfully by the model that accounted for insect survival (Fig 2). This result is an indication that additional potential complicating factors such as the insect sex ratio (which were not reported in Dubern [2]), were not a dominant influence on survival in the results. The short laboratory lifespan reported here is consistent with that reported in Chant [28] (who report that most insects enclosed on cassava plants died after 4–5 days). Few retention period assays have been reported for CMB (but see Dubern [29] in which equivalent data to that of Dubern [2] was presented). Chant [28] conducted a brief retention period assay—up to 48h—which we do not analyse here due to its brevity. The 4–5 day *B. tabaci* lifespan on cassava reported in Chant [28] raise the possibility that low lifespan is an intrinsic feature of *B. tabaci* in the laboratory, with the further decrease in survival in the present results associated with manual transfers between plants, see Table 2c).

### Intermediate peak CMV-infectiousness of *Bemisia tabaci*

We found that there was an intermediate peak in insect infectiousness near the 5th day of insect infection. This finding is important for two reasons. Firstly, given that infectiousness was increasing up to around the 5th day of infection, this constitutes further evidence favouring the conclusion that the 9d retention estimate for CMB was an underestimate of the true retention period. Secondly, a similar pattern, but with virus levels in the *B. tabaci* insect vector increasing for the first few days, has been reported for the tomato yellow leaf curl begomovirus (TYLCV) [26, 30].

For TYLCV, increasing virus levels prior to a decrease after a few days was taken as indicative of viral replication in the insect (with the decrease reflecting counteraction of viral replication by insect immune response) [26]. Indeed, when viruliferous insects were exposed to stressors to compromise their immunity, virus levels were found to increase throughout the retention period [26]. There is currently no evidence that CMB replicates in the *B. tabaci* insect host. Nevertheless, the similarity between the trajectories for TYLCV virus abundance in the insect, and insect infectiousness with CMB, is striking. This pattern may be of relevance for recent debate on whether or not begomoviruses may be whitefly pathogens [27].

Indeed, it may now be timely to individually assess published retention period experiments for a range of additional insect-borne plant pathogens—i.e. to ascertain whether or not an artefact due to low insect survival has influenced retention period estimates more widely. The role of sex ratio in the insect infectiousness trajectory is beyond the current work, as there was no available data on how *B. tabaci* sex ratio relates to infectiousness with CMB. It would be worth investigating the role of sex ratio in CMB transmission in future empirical and modelling work—and reference should be made to reported sex effects for additional *B. tabaci*-transmitted begomoviruses.

### Conclusion

Three main points pertaining to CMB virology, and a recommendation for AP experiments in general, emerge from our results. Firstly, there is no evidence that CMB retention in *B. tabaci* is less than life-long. Secondly, per capita *B. tabaci* infectiousness with CMB initially increases in the period following acquisition access. Thirdly, the existing AP evidence is more consistent with high rates of CMB acquisition by *B. tabaci*—i.e., high CMB *B. tabaci* susceptibility—as opposed to the low rates originally reported. Note that the original reports were supported by field observations of low susceptibility [31]. The field observations results, like for Dubern [2], were based upon implicit assumptions of 100% inoculation ability by whitefly and 100% survival of transfer from field conditions where the insects were gathered to glasshouse healthy test plants. Therefore the estimates for susceptibility of both Dubern [2] and [31] should only be considered as lower bounds. What do our findings mean for the large number of virology results obtained through AP experiments? If low insect survival was present in a retention period assay that involved a sequence of IAPs—then any associated estimate of retention period may significantly underestimate the true retention period.

An apparent predominance of cuttings-borne infection in the field and low insect vector transmission efficiency indicated in the laboratory have combined to downplay the importance of *B. tabaci*-borne CMB transmission. In field conditions, detailed inspection of plants have indicated that a higher proportion emanate from infected cuttings than from insect infection [12, 32]. It is therefore understandable that growers may be more concerned with CMB infection from plant cuttings than from *B. tabaci* in these situations. Epidemics of cassava mosaic disease, however, will not occur without *B. tabaci*-borne transmission (beyond small scale incursions in contaminated cuttings). Therefore it must be borne in mind that late stage epidemics for which insect-borne infection was essential, will still typically result in near total depletion of susceptible cuttings for replanting. This may give the erroneous impression that only the cuttings mode of transmission was important.

Taken together, our results indicate that individual *B. tabaci* insects are more likely to acquire CMB than previously thought, and to retain CMB for longer than previously thought. In addition, per capita *B. tabaci* infectiousness with CMB is likely to increase in the days after the insect has left an infected plant and begun to feed on healthy plants. The evidence presented here therefore showed that cassava infection from *B. tabaci* whitefly is a highly efficient mode of CMB transmission.

## Supporting information

S1 Appendix Model 1. Non-technical and technical descriptions of probability model 1.
(PDF)

S2 Appendix Hypothesis test. Description of hypothesis test and accompanying test statistic—constructed from inferences for two model 1 parameters.
(PDF)

S3 Appendix Model 2. Non-technical and technical descriptions of probability model 2.
(PDF)

S4 Appendix Probability of inoculum presence. Derivation of the probability of inoculum presence.
(PDF)
