# Supplementary material for: A new method for the analysis of access period experiments, illustrated with whitefly-borne cassava mosaic begomovirus
Source: PLoS Comput Biol. 2023 Aug 10;19(8):e1011291. doi: 10.1371/journal.pcbi.1011291 (PMC10461850; doi:10.1371/journal.pcbi.1011291)
Supplement: S1 Appendix — Non-technical and technical descriptions of probability model 1. (PDF) [file pcbi.1011291.s001.pdf]

Donnelly and Gilligan A new method for the analysis of access period experiments, illustrated with whitefly-borne cassava mosaic begomovirus

Supporting information contains details on the probability models used in the model-based analysis. S1 Appendix and S3 Appendix provide both non-technical summaries as well as technical descriptions of probability model 1 and model 2, respectively - which were used to derive the results in the main text. S2 Appendix additionally describes a hypothesis test and accompanying test statistic - constructed from inferences for two model 1 parameters. S4 Appendix provides a derivation of the probability of inoculum presence, which is used in the main text to assess the point at which the experiment became uninformative.

S1 Appendix, Model 1

Non-technical summary of Model 1

Probability model 1 consists of discrete random variables representing latent variables for the overall number of insects, and the number of infected insects, in the retention period assay (W_j total insects and W_j^I infected insects for the jth IAP). These latent variables are unobserved in the experiment but can be represented as random variables that are linked through probability parameters to the known initial insect cohort size ($W_0 = 10$) and the observed data for the number of extant insect cohorts and the number of infected plants. See schematic depiction in Fig 1 (main text) with labels for random variables and probability parameters.

Fig 1 illustrates how random variables change as the number of cohort transfers increases. For instance, the number of insects in consecutive IAPs changes according to the probability of insect death (m_0 for the initial IAP, m for subsequent IAPs). This leads to an equation for the probability that a cohort is alive in stage j (Eq S1.1 in subsequent

section). In addition, the number of infected insects that acquire the virus in the acquisition stage, out of a total of $W_0 = 10$ initial insects, depends on the probability of virus acquisition (α). In the inoculation access phase, the number of infected insects changes again in consecutive IAPs according to the probability that infected insects are lost (μ_0 for the initial IAP, μ for subsequent IAPs). The number of test plants that become infected in IAP j depends on the number of infected insects and the probability of inoculation (β). These considerations lead to an equation for the probability that a test plant in IAP j becomes infected (Eq S1.2 in subsequent section).

The strategy is to find the unknown parameter values that best fit the observed data. This means using an MCMC algorithm to sample parameter values based upon the model 1 likelihood (i.e., Eqs S1.1 and S1.2) and the summary data in Table 1, with Bayesian inference of posterior distributions for the parametrs. Parameter fits (in the form of parameter distributions) for the insect mortality parameters (i.e., m_0, m) are produced by combining the equation for the probability that an insect cohort is alive at IAP j (Eq S1.1) and the live cohort data (Table 1a). Parameter fits (in the form of parameter distributions) for the Model 1 infection parameters (α, μ_0, μ and β) are produced by combining the equation for the probability of test plant infection at IAP j (Eq S1.2) and the plant infection data (Table 1b). The Model 1 parameter distributions are summarised in Table 1A.

Bayesian inference involves the use of data (or evidence) to update a prior. In order for our study to be objective it is necessary to select non-informative prior distributions, while a natural choice for the family of the prior distribution improves computation. In this study, since the likelihood of the data given the unknown parameters ultimately follows a binomial distribution, we select $beta(1, 1)$ prior distributions for the model 1 parameters (see Table 1)(nb. the beta distribution is the conjugate prior of the binomial distribution, and $beta(1, 1)$ is equal to the non-informative uniform distribution on the $[0, 1]$ interval).

Technical summary of Model 1

There are two independent stage-specific components to model 1. The two model components correspond to the two distinct sets of data that were recorded in the retention period assay of Dubern [1]: the number of live cohorts and the number of test plant infections for each IAP. By stage-specific we are referring to an underlying equation form that accounts for daily transfers, i.e., the number of cohort transfers corresponding to a particular test plant. The first model component relates the binary data for insect cohort persistence for n replicate experiments to a simple model of insect mortality for each IAP. The second model component relates the binary data for n replicate experiments for test plant infection to a model of pathogen inoculation for each IAP.

Model component a, cohort persistence

The probability of $e_j = E_{j-1} - E_j$ cohort extinctions for $j = 2..J$ at IAP j (and of $e_1 = n - E_1$ cohort extinctions in the initial IAP), where E_j is the number of replicates with extant insects at IAP j, is described as follows. Note that initially there were 10 insects (i.e., $W_0 = 10$) and 30 replicates (i.e., $n = 30$), in the retention period assay of Dubern [1]. Accordingly, using a general formulation, the probability of e_j cohort extinctions out of a total of n reps is,

$$P(e_j|W_0, m_0, m) = q_j^{e_j} \tag{S1.1}$$

$$where\ q_j = \sum_{y=1}^{W_0} \binom{W_0}{y} w_{j-1}^{y} (1 - w_{j-1})^{W_0 - y} m^y \tag{S1.2}$$

Eq S1.1, relates the probability of extinction for an experimental replicate in stage j, denoted q_j, to two mortality parameters. These parameters are m_0, the probability of insect death in the initial stage where newly developed adults are transferred from an

infected plant to a healthy plant, and, m, the probability of insect death in all subsequent transfers. The composite parameter w_j represents the probability of individual insect survival until stage j, and is based upon initial insect survival $1 - m_0$ and subsequent survivals which increase geometrically up to the previous IAP $j - 1$, i.e., $(1 - m)^{j-1}$, so that overall survival is $w_j = (1 - m_0)(1 - m)^{j-1}$ (see Eq S1.2). Based on these parameters, q_j conditions extinction in stage j on all possible values for the number of alive insects denoted by y at the start of the IAP, which in turn defines the probability of cohort extinction in stage j given y alive insects, i.e. m^y (see Eq S1.2).

As such, in Eq S1.1, q_j represents the probability that a cohort goes extinct at stage j while the response variable $e_j = E_{j-1} - E_j$ represents the number of reps that actually went extinct at stage j in the retention period assay (Table A). Since extinct groups of insects must remain extinct in later transfers Eq S1.1 is more suitable than a model representing binomial samples from a total of n reps at each IAP.

Model component b, test plant infection

The probability of plant infection involves binomial sampling from a total of n reps. The probability of success in the binomial sampling is the probability of any inoculation event occurring (i.e., considering potential inoculation events from each insect in the cohort), i.e., $1 - p_j(0)$, in which $p_j(x)$ is the probability of x inoculations in stage j (note that p_j has several additional functional dependencies see following equations that we omit here for simplicity). See Table A for a summary of the parameters used in Model 1. In what follows we use a formulation with separate parameters for the acquisition and inoculation probabilities (α and β), but ultimately these parameters occur together as a compound parameter which we call vector efficiency ($\alpha\beta$) and therefore only vector efficiency appears in Table A. This leads to the following probability model,

$$P(R_I^j|W_0, \alpha, \beta, m_0, \mu) \sim Bin(n, 1 - p_j(y = 0, W_0, \alpha, \beta, m_0, \mu)), \tag{S1.3}$$

where,

$$p_j(y) = \overbrace{\sum_{y=0}^{W_0} \binom{W_0}{h} \alpha^h (1-\alpha)^{10-h}}^{\text{acquisition}} \overbrace{\sum_{k=0}^{h} \binom{h}{k} \nu_{j-1}^k (1-\nu_{j-1})^{y-k}}^{\text{survival}} \overbrace{\sum_{y=0}^{k} \binom{k}{y} \beta^y (1-\beta)^{k-y}}^{\text{inoculation}} \tag{S1.4}$$

$$= \sum_{y=0}^{W_0} \binom{W_0}{y} (\alpha\nu_{j-1}\beta)^y (1 - \alpha\nu_{j-1}\beta)^{W_0 - y} \tag{S1.5}$$

in which, $\nu_j = (1 - m_0)(1 - \mu)^{j-1}$ is the probability that individual infectious insects remain infectious until stage j. In turn, ν_j depends on the following assay parameters: μ_0, the probability of loss of infection in the initial stage where newly developed adults are transferred from an infected plant to a healthy plant, and, μ, the probability of loss of infection in all subsequent IAPs. In addition, α is the probability that an individual acquires the pathogen during acquisition access, and β is the probability that an individual infected insect inoculates the pathogen into the healthy plant during inoculation access. Note that Eq S1.5 is obtained by simplifying Eq S1.4 using the binomial property that conditional binomials combine to form another binomial.

Since,

$$p_j(y = 0, W_0, \alpha\beta, m_0, \mu) = (1 - \nu_{j-1}\alpha\beta)^{W_0}, \tag{S1.6}$$

the probability of R_I^j infected test plants in the assay in the jth IAP is,

$$P(I_j|W_0, n, \alpha\beta, m_0, \mu,) \sim Bin\Big(n_1, 1-(1-\alpha\nu_{j-1}\beta)^{W_0}\Big), \tag{S1.7}$$

104 with $\nu_{j-1} = (1-m_0)(1-\mu)^{j-1}$.

A) Model 1	***Parameter to be fitted***	***Prior distribution***
m_0	Prob. of insect death, initial transfer	$\sim beta(1,1)$
m	Prob. of insect death, subsequent transfers	$\sim beta(1,1)$
μ	Prob. of infected insect loss, subsequent transfers	$\sim beta(1,1)$
$\alpha\beta$	Vector efficiency (acquisition prob. $\times$ inoculation prob.)	$\sim beta(1,1)$
**B)**	***Derived parameter***	
q_j	Prob. of cohort extinction, j^{th} IAP	
ω_j	Prob. insect survives to stage j	
ν_j	Prob. infected insect remains to stage j	
$p_j(y)$	Prob. of y inoculation events, IAP j	
**C)**	***Data variables***	
n	# Experiment replicates	pre-existing data
E_j	# Replicate cohorts with live insects, j^{th} IAP	pre-existing data
I_j	# Replicate cohorts with test plant infection, j^{th} IAP	pre-existing data
e^j	# cohorts that became extinct, j^{th} IAP	pre-existing data
W_0	initial insect cohort size	pre-existing data
J	total no. of IAPs	pre-existing data

Table A: Parameter definitions, and prior distributions, for the Model 1 Bayesian analysis. Derived parameters are combinations of fitted parameters in A). All prior distributions were chosen to be non-informative and with an initial condition of 10^{-4}. Posterior parameter distributions were obtained using Hamiltonian Monte Carlo, RStan version 2.21.0 [2], R version 3.6.3 [3]. Additional rStan settings were: $warmup = 400$, $chains = 4$, $iterations = 2000$, $max(treedepth) = 15$, $adapt\_delta = 1 - (10^{-1})$.

REFERENCES

1. Dubern J. (1994) Transmission of African cassava mosaic geminivirus by the whitefly (Bemisia tabaci). *Tropical Science*, 34(1):82-91.

2. Stan Development Team. (2022) *R: A language and environment for statistical computing*, in: R Foundation for Statistical Computing, Vienna, Austria, *RStan: the R interface to Stan*, R package version 2.21.7 Available at: https://mc-stan.org/

3. Core team. (2014) *R: A language and environment for statistical computing*, in: R Foundation for Statistical Computing, Vienna, Austria, Available at: http://www.R-project.org/
