# Supplementary material for: A new method for the analysis of access period experiments, illustrated with whitefly-borne cassava mosaic begomovirus
Source: PLoS Comput Biol. 2023 Aug 10;19(8):e1011291. doi: 10.1371/journal.pcbi.1011291 (PMC10461850; doi:10.1371/journal.pcbi.1011291)
Supplement: S2 Appendix — Description of hypothesis test and accompanying test statistic—constructed from inferences for two model 1 parameters. (PDF) [file pcbi.1011291.s002.pdf]

S2 Appendix, Hypothesis test

We developed a hypothesis test to investigate the null hypothesis representing life-long retention of a given pathogen in the insect vector. A suitable test statistic is the relative size of the mortality rate of the insect vector and the rate at which infected insects are lost (which can be through pathogen clearance or vector mortality). The test statistic can be calculated from the Model 1 parameter fits as follows. The ratio distribution of the parameter distributions for the probability of infected insect loss (μ) and for the probability of insect mortality (m) can be computed using the MCMC algorithm.

The parameter distribution representing the ratio of the two parameter distributions forms a test statistic. This statistic is then tested for significant deviation from unity which would indicate a non-zero recovery probability. Note that the hypothesis test is a means to investigate the alternative hypothesis that infected insect loss - which encompasses both insect death and pathogen clearance - occurs more rapidly than death (i.e. a less than life-long retention period).
