# Supplementary material for: A new method for the analysis of access period experiments, illustrated with whitefly-borne cassava mosaic begomovirus
Source: PLoS Comput Biol. 2023 Aug 10;19(8):e1011291. doi: 10.1371/journal.pcbi.1011291 (PMC10461850; doi:10.1371/journal.pcbi.1011291)
Supplement: S3 Appendix — Non-technical and technical descriptions of probability model 2. (PDF) [file pcbi.1011291.s003.pdf]

S3 Appendix, Model 2

Non-technical summary of Model 2

In this appendix we modify the main model (see Appendix S1) to incorporate variation in insect infectiousness across the daily transfers of the retention period assay. The modified model (model 2) is useful for addressing a lack of statistical identifiability for insect infectiousness in the underlying models (see Box 1 main text for an explanation of this statistical term). In essence, insects do not lose the infection in the model 2 representation but their infectiousness to healthy test plants is allowed to change as the number of daily transfers increases. Note that since this representation allows for zero infectiousness and is otherwise the same as model 1, the two models are equivalent. In brief, a Bayesian approach using the modified model is used to infer posterior distributions for percentage peak infectiousness as the number of daily transfers increases. While different from insect infectiousness, distributions for percentage peak infectiousness may be a sufficient measure of insect infectiousness. The method that we used to achieve this is summarised in Box 1 main text and requires additional inference of peak vector efficiency (the distribution for the maximum vector efficiency across the IAPs of the experiment, where vector efficiency is the product of the probability of pathogen acquisition in the AAP and the probability of pathogen inoculation in the IAP for a given day-long IAP). Following the same rationale outlined in S1 Text, we select $beta(1,1)$ prior distributions for the model 2 parameters (see Table A in S3 Text).

Technical description of Model 2

Following the model 1 steps (S1 Text), but allowing the probability of inoculation in each IAP to differ (i.e., β_j), leads to the following probability model for plant infection is,

$$P(I_j|W_0, n, \alpha\beta_j, m_0, m) \sim Bin\Big(n, 1 - (1 - \omega_{j-1}\alpha\beta_j)^{W_0}\Big), \tag{S3.1}$$

with $\omega_{j-1} = (1 - m_0)(1 - m)^{j-1}$. Note that the survival component ω_{j-1} depends only on mortality since there is no pathogen clearance. Again, α and β_j are not separable at the outset. A posterior parameter distribution for % peak infectiousness, however, can be inferred from $\alpha\beta_j$ if we divide by the peak infectiousness posterior distribution, i.e.,

$$\textbf{Percentage peak infectiousness} \quad \frac{\beta_j}{\beta^{peak}} = \frac{\alpha\beta_j}{\alpha\beta^{peak}} \tag{S3.2}$$

The vector efficiencies specific to IAP are summarised for reference in Table B and shown in Fig A. See Table A for a summary of the parameters used in Model 2.

A)	Model 2	***Parameter to be fitted***	***Prior distribution***
	m_0	Prob. of insect death, initial IAP	$\sim beta(1,1)$
	m	Prob. of insect death, subsequent IAPs	$\sim beta(1,1)$
	$\alpha\beta_j$	Stage j vector efficiency	$\sim beta(1,1)$
**B)**		***Derived parameter***	
	ω_j	Prob. insect survives to stage j	
	$p_j(y)$	Prob. of y inoculation events, IAP j	
	$\alpha\beta^{peak}$	Peak vector efficiency	

Table A: Parameter definitions, and prior distributions, for the Model 2 Bayesian analysis. Derived parameters are combinations of fitted parameters in A). See Table A in S1 Appendix for data variable summary and see caption of Table S1.2 for technical details.

Vector efficiency	Median	2.5%	97.5%	% Peak infectiousness	Median	2.5%	97.5%
$\alpha\beta_1$	0.494	0.270	0.861	$\hat{\beta}_1 = 100 \times \beta_1/\beta^{peak}$	54.6	29.7	95.5
$\alpha\beta_2$	0.457	0.261	0.752	$\hat{\beta}_2 = 100 \times \beta_2/\beta^{peak}$	50.4	28.7	83.4
$\alpha\beta_3$	0.477	0.279	0.805	$\hat{\beta}_3 = 100 \times \beta_3/\beta^{peak}$	52.4	30.6	88.2
$\alpha\beta_4$	0.705	0.420	0.966	$\hat{\beta}_4 = 100 \times \beta_4/\beta^{peak}$	77.8	46.3	100.0
$\alpha\beta_5$	0.848	0.546	0.993	$\hat{\beta}_5 = 100 \times \beta_5/\beta^{peak}$	95.3	62.4	100.0
$\alpha\beta_6$	0.727	0.391	0.982	$\hat{\beta}_6 = 100 \times \beta_6/\beta^{peak}$	80.6	43.8	100.0
$\alpha\beta_7$	0.676	0.323	0.975	$\hat{\beta}_7 = 100 \times \beta_7/\beta^{peak}$	74.6	36.1	100.0
$\alpha\beta_8$	0.407	0.111	0.911	$\hat{\beta}_8 = 100 \times \beta_8/\beta^{peak}$	44.8	12.0	99.5
$\alpha\beta_9$	0.557	0.156	0.971	$\hat{\beta}_9 = 100 \times \beta_9/\beta^{peak}$	61.7	17.6	100.0
$\alpha\beta_{10}$	0.375	0.054	0.938	$\hat{\beta}_{10} = 100 \times \beta_{10}/\beta^{peak}$	41.5	5.9	100.0
$\alpha\beta_{11}$	0.221	0.008	0.877	$\hat{\beta}_{11} = 100 \times \beta_{11}/\beta^{peak}$	24.4	0.95	95.8
$\alpha\beta_{12}$	0.287	0.009	0.941	$\hat{\beta}_{12} = 100 \times \beta_{12}/\beta^{peak}$	31.8	1.0	100.0
$\alpha\beta_{13}$	0.351	0.013	0.946	$\hat{\beta}_{13} = 100 \times \beta_{13}/\beta^{peak}$	38.7	1.4	100.0

Table B: Parameter estimates for insect vector efficiency and percentage peak vector efficiency by IAP in a Bayesian analysis of Dubern [1]'s retention period assay. See caption of Table 3 main text for further details.

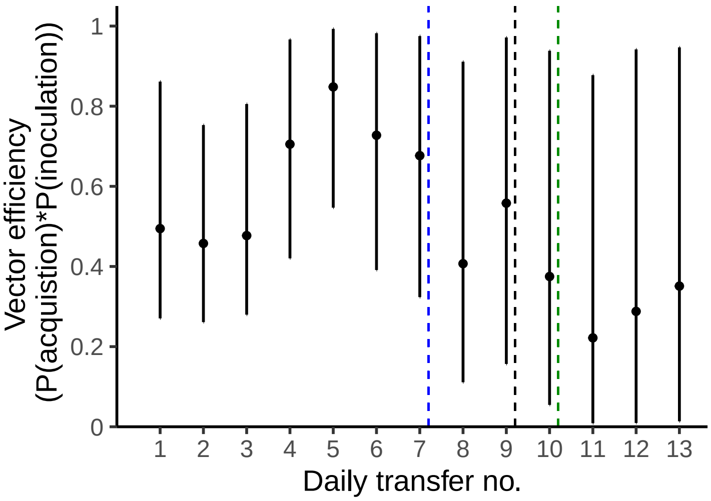

Figure A: Insect vector efficiency in multiple consecutive IAPs in a retention period assay. Median and 95% credible intervals for model assay output are represented throughout with black circles and vertical black bars respectively. The 2.5th, 50th and 97.5th percentiles (i.e., 95% credible intervals and median values) for the model assay output were generated from the parameter estimates in Table 4 (Model 2) in B. See caption of Fig 2 for further technical details.

REFERENCES

1. Dubern J. (1994) Transmission of African cassava mosaic geminivirus by the whitefly (Bemisia tabaci). *Tropical Science*, 34(1):82-91.
