# Supplementary material for: A new method for the analysis of access period experiments, illustrated with whitefly-borne cassava mosaic begomovirus
Source: PLoS Comput Biol. 2023 Aug 10;19(8):e1011291. doi: 10.1371/journal.pcbi.1011291 (PMC10461850; doi:10.1371/journal.pcbi.1011291)
Supplement: S4 Appendix — Derivation of the probability of inoculum presence. (PDF) [file pcbi.1011291.s004.pdf]

S4 Appendix, Probability of inoculum presence

In this appendix we derive the equation underlying the calculation of the probability that inoculum was present in the experiment in IAP j, denoted σ_j. Firstly, the probability that an infected insect is lost by stage j, denoted η_j, depends upon the sum of the probabilities of infected vector loss up to and including stage j. In stage 1, this is simply m_0. In stage 2, this is $m_0 + (1 - m_0)\mu$. In stage 3, this is $m_0 + (1 - m_0)\mu + (1 - m_0)(1 - \mu)\mu$ and so on up to $j = J$. Accordingly,

$$\eta_1 = m_0 \qquad for\ j = 1, \tag{S4.1}$$

$$\eta_j = m_0 + \sum_{y=2}^{J} m_0(1-\mu)^{y-2}\mu \qquad for\ j = 2..J. \tag{S4.2}$$

Secondly, the probability that no inoculum is present in a stage j replicate, denoted δ_j, depends upon the binomially distributed number of insects out of a total of W_0 that successfully acquired the pathogen in the acquisition stage, multiplied by the probability that all such insects lost the pathogen or died by stage j, i.e.,

$$\delta_j = \sum_{x=1}^{W_0} \binom{W_0}{x} \alpha^x (1-\alpha)^{W_0-x} \eta_j^x. \tag{S4.3}$$

Finally, for inoculum to be entirely absent in the experiment in stage j, there must be no inoculum present in each stage j replicate. Accordingly,

Probability of stage j inoculum presence $$\sigma_j = 1 - \delta_j^n. \tag{S4.4}$$

Note that the number of replicates was $n = 30$ in Dubern [1] and the initial cohort size was $W_0 = 10$. Eq S4.4 is used to generate the curves that appear in Fig 3b, main text. Distinct curves are shown for different values of the probability of acquisition, α, since this parameter appears in Eq S4.4 but has not been inferred in our results (blue and green dots represent median values if $\alpha = 0.25$ or $\alpha = 0.75$ respectively; black dots and bars represent median values and 95% credible intervals respectively if $\alpha = 0.5$). For instance, we find that by IAP 7 (red vertical dashed line) it was no longer possible to be 95% confident (red horizontal dashed line represents $\nu = 0.95$) that there was any inoculum present in any of the experimental replicates if $\alpha = 0.5$ (note that if $\alpha = 0.1$ this point was reached earlier at around IAP 4 while if $\alpha = 0.9$ this occurred later at around IAP 10).

REFERENCES

1. Dubern J. (1994) Transmission of African cassava mosaic geminivirus by the whitefly (Bemisia tabaci). *Tropical Science*, 34(1):82-91.
